# Washed microbiota transplantation vs. manual fecal microbiota transplantation: clinical findings, animal studies and *in vitro* screening

**DOI:** 10.1007/s13238-019-00684-8

**Published:** 2020-01-09

**Authors:** Ting Zhang, Gaochen Lu, Zhe Zhao, Yafei Liu, Quan Shen, Pan Li, Yaoyao Chen, Haoran Yin, Huiquan Wang, Cicilia Marcella, Bota Cui, Lei Cheng, Guozhong Ji, Faming Zhang

**Affiliations:** 1 Medical Center for Digestive Diseases, The Second Affiliated Hospital of Nanjing Medical University, Nanjing, 210011 China; 2 Key Lab of Holistic Integrative Enterology, Nanjing Medical University, Nanjing, 210011 China; 3 Tianjin Key Laboratory of Optoelectronic Detection Technology and Systems, Tianjin, 300387 China; 4 Department of Microbiology, School of Medicine, Jiangsu University, Zhenjiang, 212013 China; 5 Biogas Institute of Ministry of Agriculture and Rural Affairs, Chengdu, 610041 China; 6 Center for Anaerobic Microbial Resources of Sichuan Province, Chengdu, 610041 China; 7 Division of Microbiotherapy, Sir Run Run Shaw Hospital, Nanjing Medical University, Nanjing, 211166 China

**Keywords:** fecal microbiota transplantation, washed microbiota transplantation, adverse event, safety, infection, virus, metabolomics, spectroscopy, transplant

## Abstract

Fecal microbiota transplantation (FMT) by manual preparation has been applied to treat diseases for thousands of years. However, this method still endures safety risks and challenges the psychological endurance and acceptance of doctors, patients and donors. Population evidence showed the washed microbiota preparation with microfiltration based on an automatic purification system followed by repeated centrifugation plus suspension for three times significantly reduced FMT-related adverse events. This washing preparation makes delivering a precise dose of the enriched microbiota feasible, instead of using the weight of stool. Intraperitoneal injection in mice with the fecal microbiota supernatant obtained after repeated centrifugation plus suspension for three times induced less toxic reaction than that by the first centrifugation following the microfiltration. The toxic reactions that include death, the change in the level of peripheral white blood cells, and the proliferation of germinal center in secondary lymphoid follicles in spleen were noted. The metagenomic next-generation sequencing (NGS) indicated the increasing types and amount of viruses could be washed out during the washing process. Metabolomics analysis indicated metabolites with pro-inflammatory effects in the fecal microbiota supernatant such as leukotriene B4, corticosterone, and prostaglandin G2 could be removed by repeated washing. Near-infrared absorption spectroscopy could be served as a rapid detection method to control the quality of the washing-process. In conclusion, this study for the first time provides evidence linking clinical findings and animal experiments to support that washed microbiota transplantation (WMT) is safer, more precise and more quality-controllable than the crude FMT by manual.

**Electronic supplementary material:**

The online version of this article (10.1007/s13238-019-00684-8) contains supplementary material, which is available to authorized users.

## Introduction

Fecal microbiota transplantation (FMT), an effective method of reconstructing the overall gut microbiota of patients, has a wide range of therapeutic indications (Zhang et al., 2018; Allegretti et al., 2019). It has been recommended for the treatment of recurrent *Clostridioides difficile* infection (CDI) in the clinical guideline, consensus and joint-experts recommendation (McDonald et al., 2018; Ng et al., 2019). Increasing published randomized controlled trials (RCTs) and real-world studies demonstrated that FMT is of important therapeutic value in diseases beyond CDI, such as ulcerative colitis (UC) (Moayyedi et al., 2015; Paramsothy et al., 2017; Costello et al., 2019; Ding et al., 2019; Sood et al., 2019), Crohn’s disease (CD) (Wang et al., 2018), hepatic encephalopathy (Bajaj et al., 2017), and autism (Kang et al., 2017). Tracing the origins of FMT in the world medical history, the earliest record of FMT for the treatment of human diseases began at least in the 4th century in China (Zhang et al., 2012). However, up to now, the protocol introduced in published consensuses is merely mixing the stool with saline in a blender by the manual method (Cammarota et al., 2017; Konig et al., 2017; Ng et al., 2019). The fecal matter actually includes many particles that even caused obstruction through colonic transendoscopic enteral tube (Wang et al., 2019). The dose for delivering FMT is mainly based on the donors’ fecal weight, instead of the precise volume or amount of microbiota (Cammarota et al., 2019).

The recent surveys on doctors, medical students, donors, and patients show that they have a negative perception towards FMT, especially its crude methods (Zipursky et al., 2014; Ma et al., 2017; Park et al., 2017; McSweeney et al., 2019; Wu et al., 2019). Doctors are generally less willing to recommend FMT than patients, and one reason is that they need to have contact with feces and prepare the feces by manual method (Brandt, 2012; Zipursky et al., 2012, 2014; Ma et al., 2017). Based on a survey of 241 patients, 22% think it is “dirty/unsanitary”, and this is a possible reason why they might not be willing to receive FMT (Park et al., 2017). Negative perceptions of society significantly limit the use of FMT. Importantly, DeFilipp et al. (2019) recently reported that two patients developed extended-spectrum beta-lactamase (ESBL)-producing bacteremia after FMT. This highlighted the importance to improve the safety of FMT. Therefore, the FMT-standardization Study Group stated that it is time to leave the manual FMT behind and move FMT standardization forward (Zhang et al., 2018).

An automatic preparation method for enriching microbiota from feces has been developed and is being used in serial FMT centers in China since 2014 (Cui et al., 2015; Cui et al., 2016; Qi et al., 2018; Wang et al., 2018; Ding et al., 2019; Huang et al., 2019). This method, originally designed on the concept of washed microbiota preparation is based on the automatic microfiltration machine (GenFMTer, Nanjing, China) and the following repeated centrifugation plus suspension with support from specific facilities. Based on the patients who underwent either washed microbiota transplantation (WMT) or crude FMT in the same FMT center with the same clinical workflow, the multiple factors analysis demonstrated that the washed microbiota preparation is an independent factor contributing to the decreased adverse events (AEs) from 38.7% to 14.4% in patients with UC(Ding et al., 2019). The rate of AEs decreased significantly from 21.7% in crude FMT to 8.7% in WMT in patients with CD (Wang et al., 2018). Importantly, the washed microbiota preparation did not affect the efficacy in both populations with UC and CD, compared with that by manual preparation for fecal microbiota (Wang et al., 2018; Ding et al., 2019).

However, there is lacking reported theoretical evidence for understanding the reason why washed microbiota preparation is safer and better than the commonly used crude method for fecal microbiota preparation. This study aimed to investigate how the washing preparation is better than the manual preparation for FMT.

## Results

### Washed microbiota preparation decreased the FMT-related AEs

**Figure 1:**
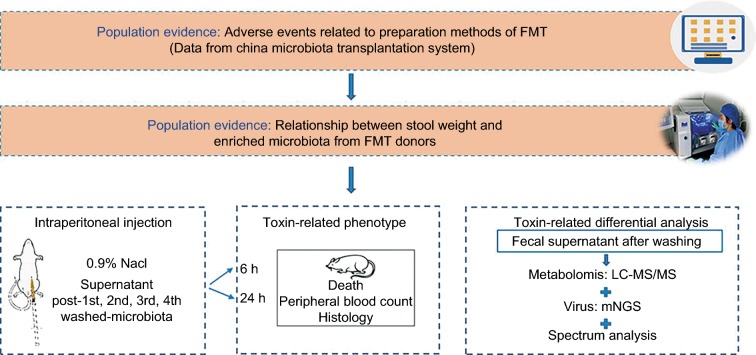
Flow chart of the study

**Figure 2:**
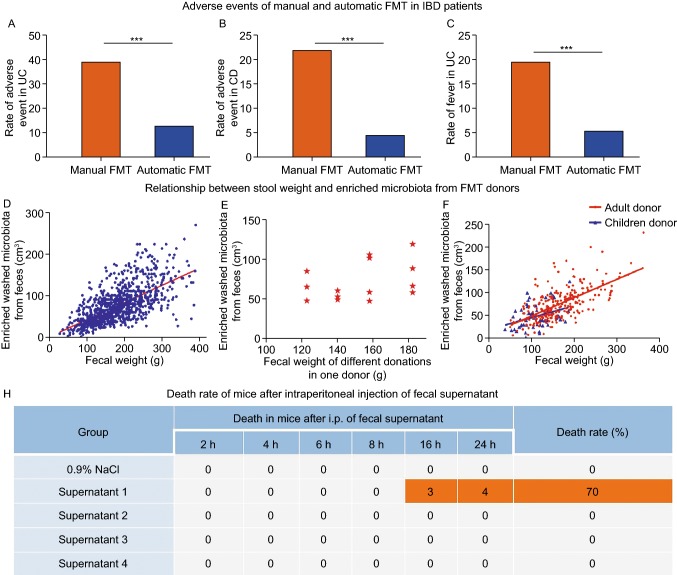
Evidence related to fecal microbiota preparation from human population to animal. (A–C) AEs related to manual and automatic preparation for fecal microbiota in patients. (D–F) Relationship between fecal weight and enriched microbiota from FMT donors. (H) Death time and rate after intraperitoneal injection of fecal supernatant in mice (i.p., intraperitoneal injection). Statistical comparisons are performed using chi-square test; *P < 0.05, **P < 0.01, ***P < 0.001. Correlation analysis was performed using Spearman correlation analysis. Data are presented as mean ± standard deviation (SD)

Clinical data from a total of 970 patients (478 with UC and 492 with CD) who underwent FMT was recorded into China microbiota transplantation system (CMTS) for over a one-year follow-up (Fig. 1). Figure 2 showed the rate of AEs decreased significantly in patients with UC who underwent an automatic method for the preparation of fecal microbiota than those who experienced a manual method (38.7% vs. 12.3%, *P* < 0.001). In CD, the rate of AEs in patients who underwent a manual method was 21.7%, which was significantly higher than the rate of 4.26% in those who underwent an automatic method (*P* < 0.001). The fever after FMT significantly decreased from 19.35% in manual preparation to 5.15% in automatic preparation for fecal microbiota (*P* = 0.001).

### The relationship between fecal weight and the amount of enriched microbiota in donors

Following the automatic preparation for washing microbiota, we also explored the relationship between fecal weight and the amount of enriched microbiota. As shown in Fig. 2D, the fecal weight was correlated with the amount of enriched washed microbiota (95% CI, 0.61–0.68, *P* < 0.0001), but r was 0.65. Even for the different defecation samples from the same donor, the relationship between the fecal weight and amount of microbiota was not well satisfactory (Fig. 2E). The correlation coefficient r in adult donors is 0.65 (95% CI, 0.57–0.72, *P* < 0.0001), while it is 0.36 in children donors (95% CI, 0.10–0.57, *P* = 0.0085). The dose of enriched microbiota for adult patients and children over than 7 years is 50 cm^3^ of microbiota precipitation for the regular treatment and the dose of enriched microbiota for children from 1 to 7 year-old ranged 10–50 cm^3^. The volume ratio of final precipitation/vector solution is 1:2 for making suspension as fresh use or frozen use. For the convenience in communications during medical practice, we defined one unit to replace 10 cm^3^ microbiota precipitation (~1.0 × 10^13^ bacteria) in the medical record in CMTS and clinical flow. This measurement was confirmed in our previous experiments and it is the first time to open to public.

### Toxic responses in mice caused by intraperitoneal injection of fecal microbiota supernatant

To evaluate the role of clinical laboratory washing process on reducing the rate of AEs after WMT, the different supernatants obtained after one to four times of washing were used for intraperitoneal injection in mice. As shown in Fig. 2H, the death rate in the group of Supernatant 1 was 70%, and no death was observed in the groups of Supernatant 2, Supernatant 3, and Supernatant 4 at 24 h after intraperitoneal injection. Decreased activity, lower temperature, and chills were observed in the group of Supernatant 1, but no similar symptoms were observed in other groups.

#### Fecal microbiota supernatant induced peripheral blood cells changes at 6 h after intraperitoneal injection

**Figure 3:**
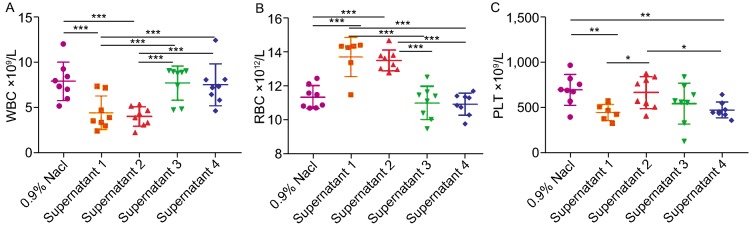
Changes of peripheral blood cells in the five groups of mice after 6 h of intraperitoneal injection of fecal microbiota supernatant. (A) Changes of WBC (*n* = 8 animals/group). (B) Changes of RBC (*n* = 8 animals/group). (C) Changes of PLT (*n* = 8 animals/group). Statistical comparisons are performed using one-way ANOVA; *P < 0.05, **P < 0.01, ***P < 0.001. Data are presented as mean ± SD

After 6 h of intraperitoneal injection, the number of white blood cells (WBC) in the group of Supernatant 1 and 2 was significantly decreased compared with the group of normal saline (*P* < 0.001, *P* < 0.001, respectively) (Fig. 3). However, the number of WBC in the Supernatant 3 was close to that in the normal saline group (7.70 ± 1.87 vs. 7.90 ± 2.11, *P* > 0.05). No significant difference was observed in the WBC count between the Supernatant 3 and Supernatant 4 group.

Among all four groups, the changes in red blood cells (RBC) are consistent with the trend of WBC (Fig. 3). There was no difference in the number of RBC between the Supernatant 3 and the normal saline group. Similarly, no significant difference was observed in both RBC and platelets (PLT) between Supernatant 3 and Supernatant 4. Figure S1 showed that neutrophil (NEUT), percentage of NEUT, lymphocyte (LYM), percentage of LYM, NEUT to LYM ratio (NLR), and PLT to LYM ratio (PLR) of the Supernatant 3 were all close to those in the normal saline group.

#### Fecal microbiota supernatant induced peripheral blood cells changes at 24 h after intraperitoneal injection

**Figure 4:**
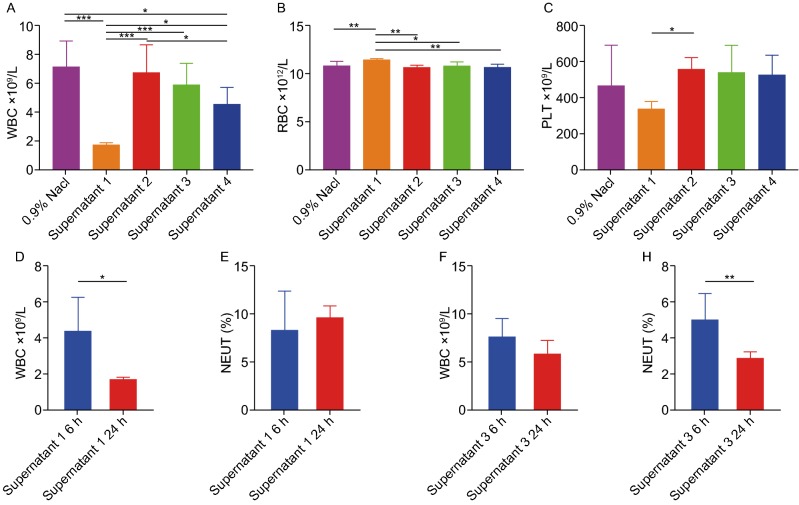
Changes of peripheral blood cells of mice after intraperitoneal injection of fecal microbiota supernatant. (A) Changes of WBC in the five groups of mice after 24 h (Supernatant 1, *n* = 3; other groups, *n* = 8). (B) Changes of RBC in the five groups of mice after 24 h (Supernatant 1, *n* = 3; other groups, *n* = 8). (C) Changes of PLT in the five groups of mice after 24 h (Supernatant 1, *n* = 3; other groups, *n* = 8). (D) Changes of WBC at 6 h and 24 h after injection with Supernatant 1 (Supernatant 1 6 h, *n* = 8; Supernatant 1 24 h, *n* = 3). (E) Changes in the percentage of NEUT at 6 h and 24 h after injection with Supernatant 1 (Supernatant 1 6 h, *n* = 8; Supernatant 1 24 h, *n* = 3). (F) Changes of WBC at 6 h and 24 h after injection with Supernatant 3 (*n* = 8/group). (H) Changes in the percentage of NEUT at 6 h and 24 h after injection with Supernatant 3 (*n* = 8/group). Statistical comparisons (A–C) are performed using one-way ANOVA; statistical comparisons (D–H) are performed using unpaired *t*-tests; *P < 0.05, **P < 0.01, ***P < 0.001. Data are presented as mean ± SD

After 24 h of intraperitoneal injection, the number of WBC in the group of Supernatant 1 was significantly lower, while the number of RBC was higher than that in the group of the normal saline (*P* < 0.001, *P* < 0.01, respectively). But no significant difference was observed between the Supernatant 3 and the normal saline group for WBC, RBC, PLT, NEUT, percentage of NEUT, NLR, LYM and PLR (Fig. 4 and S2). At the same time, there was no difference in the number of WBC, RBC and PLT between the Supernatant 3 and Supernatant 4 group.

Comparison between different time points showed that the number of WBC at 24 h was significantly lower than that at 6 h after intraperitoneal injection of the Supernatant 1 (1.74 ± 0.1 vs. 4.43 ± 1.87, *P* = 0.04) (Fig. 4). However, no significant decrease in WBC at 24 h was observed for Supernatant 3. Compared with the value at 6 h, NEUT% significantly decreased at 24 h in the Supernatant 3 group (*P* = 0.009). However, NEUT% in the Supernatant 1 group did not differ significantly between 6 h and 24 h.

#### Fecal microbiota supernatant-caused pathological change of spleen in mice

**Figure 5:**
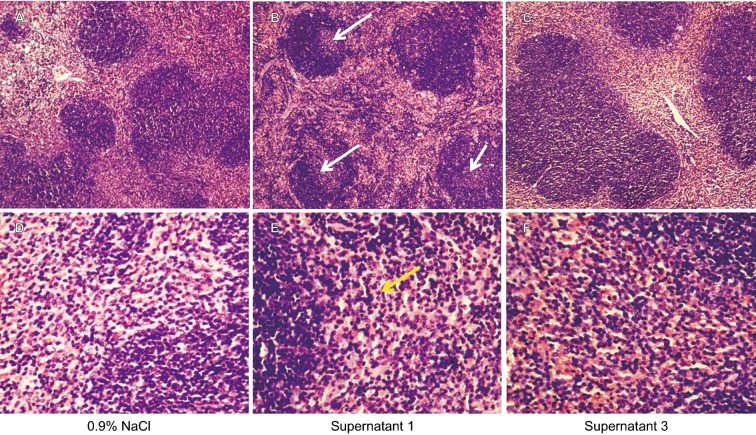
HE staining of spleen in mice at 6 h after intraperitoneal injection with the fecal microbiota supernatant. (A) No proliferation of primary follicles was observed in the group of normal saline (×200). (B) The proliferation of secondary follicles was observed in the group of Supernatant 1 (×200). (C) No proliferation of primary follicles was observed in the group of Supernatant 3 (×200). (D) NEUT infiltration in the group of normal saline (×400). (E) NEUT infiltration in the group of Supernatant 1 (×400). (F) NEUT infiltration in the group of Supernatant 3 (×400) . White arrow noted: germinal center of secondary follicles; yellow arrow noted: NEUT infiltration

As shown in Fig. 5, in the group of Supernatant 1, we observed apparent neutrophils infiltration, which was marked as “++−+++”. And multiple germinal centers of secondary lymphoid follicles were seen in the group of Supernatant 1 and the proliferation of secondary lymphoid follicles was marked as “+”. In the group of Supernatant 3, the degree of neutrophils infiltration was “+−++”, and there was no proliferation of primary follicles (−). No cavity or necrosis of spleen tissue and bacterial mass was observed in all groups.

### Differential screening of virus changes during the washing process

**Figure 6:**
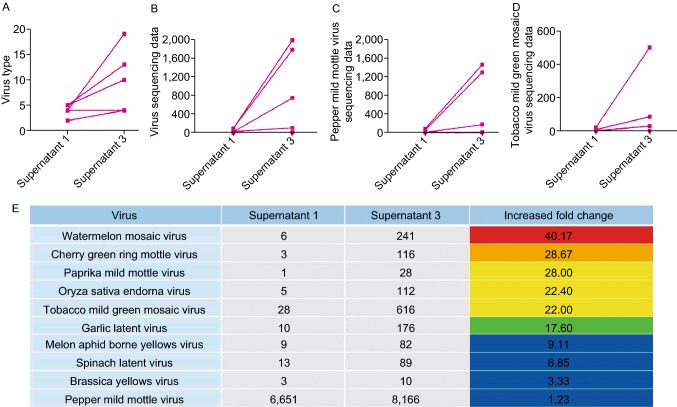
Differential screening of virus changing during the washing process. (A) Changes of virus types. (B) Changes of metagenomic NGS reads number. (C and D) Changes of the same virus in the washed fecal supernatant. (E) Top ten viruses with the most significant fold change

In order to identify what viruses were washed out during washing process, the metagenomic next-generation sequencing (NGS) was used to differentiate the Supernatant 1 and the Supernatant 3. Compared with the Supernatant 1, the types and number of viruses tested by metagenomic NGS of the Supernatant 3 showed an incremental trend (Fig. 6). Only one type of virus named pepper mild mottle virus was found in all five donors, and Tobacco mild green mosaic virus was found in four donors. The NGS reads of the Supernatant 3 also increased in these two viruses (Fig. 6C and 6D). Figure 6E showed the fold change between the Supernatant 1 and the Supernatant 3 in the number of the top ten viruses. The fold change of the virus named watermelon mosaic virus ranked the top 1, and its sequencing number in the Supernatant 3 was 40.17 times higher than that in the Supernatant 1.

**Figure 7:**
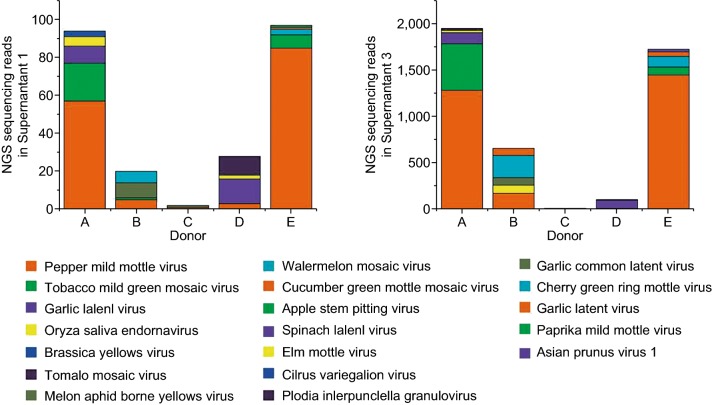
Top five viruses in each donor

Figure 7 showed the types and numbers of the top five viruses in each donor. Donor C had the minimum virus sequencing reads both in the Supernatant 1 and the Supernatant 3. Donor E had the maximum virus sequencing reads in the Supernatant 1, and Donor A had the maximum one in the Supernatant 3.

### Differential screening of metabolites during the washing process

**Figure 8:**
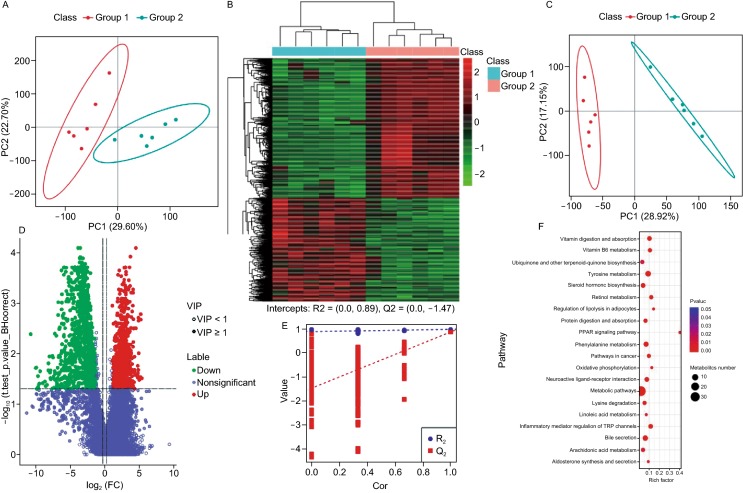
Differential screening of metabolomics during the washing process. Group 1: Supernatant 1; Group 2: Supernatant 3. (A) A score map of the PCA model. (B) Cluster analysis of differential metabolites. Each row represented a differential metabolite with each column representing a sample, and the color from green to red corresponding expression from low to high. (C) A score map of the PLS-DA model. (D) Volcanic map of differential metabolites. The down-regulated differential metabolites were labeled green, the up-regulated ones were marked in red, and the undifferentiated metabolites were labeled as purple-grey. (E) The response ranking check chart of the PLS-DA model. (F) Bubble map of metabolic pathway enrichment analysis. The X-axis enrichment factor (RichFactor) is the value of differential metabolites annotated to the pathway divided by all identified metabolites annotated to the pathway. The larger value indicated the greater proportion of differential metabolites annotated in the pathway. The dot size represented the number of differential metabolites annotated to the pathway

In order to identify what metabolites were washed out during washing process, the method of LC-MS was used to differentiate the Supernatant 1 and the Supernatant 3. The principle component analysis (PCA) and partial least squares method-discriminant analysis (PLS-DA) showed that there was a significant difference in overall metabolite composition between Supernatant 1 and Supernatant 3. A total of 78 differential metabolites were identified. Compared with the Supernatant 3, 31 differential metabolites were significantly up-regulated and 47 differential metabolites were significantly down-regulated in the Supernatant 1 (q < 0.05). The up-regulated metabolites in the Supernatant 1 included leukotriene B4 (LtB4), corticosterone (CORT), prostaglandin G2 (PGG2), 5-hydroxyindole-3-acetic acid (5-HIAA) and so on. And the metabolites of tretinoin, stearidonic acid, caffeic acid, tyrosol, aspirin, 4-hydroxy-2-nonenal, resveratrol, chloral hydrate, paracetamol, calcitriol, adenosine, etc. were up-regulated in the Supernatant 3. The results of cluster analysis of differential metabolites were shown in Fig. 8B. Based on the Kyoto encyclopedia of genes and genomes (KEGG) database, the significantly enriched metabolic pathways on differential metabolites were shown in Fig. 8F, such as inflammatory mediator regulation of transient receptor potential (TRP) channels, PPAR signaling pathway, phenylalanine metabolism (*P* < 0.05).

### Differential testing by near-infrared absorption (NIRS) spectroscopy

**Figure 9:**
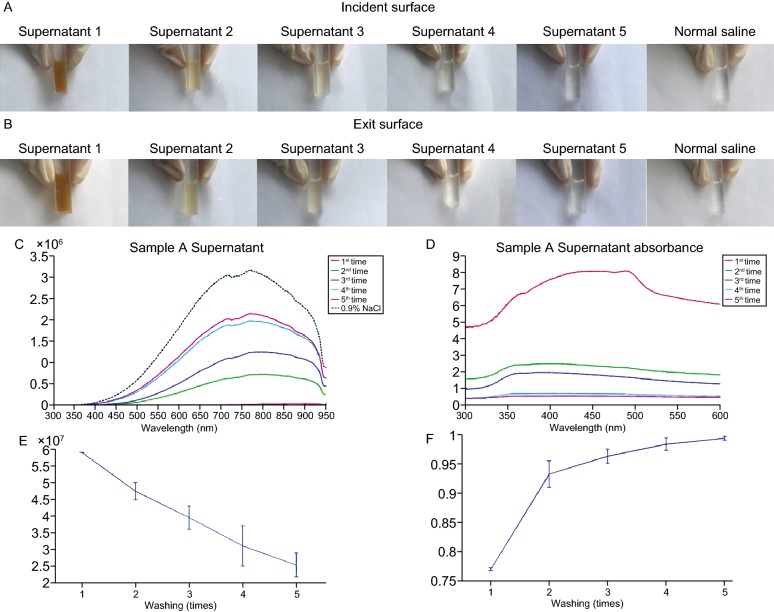
Differential testing by near-infrared absorption (NIRS) spectroscopy. (A) Incident surface of Supernatant 1, 2, 3, 4, 5 and normal saline. (B) Exit surface of Supernatant 1, 2, 3, 4, 5 and normal saline. (C) The light intensity of fecal microbiota supernatant by near-infrared absorption spectroscopy. (D) The absorbance of fecal microbiota supernatant by near-infrared absorption spectroscopy. (E) Euclidean distance between the spectrum of the supernatant of different washing times and the spectrum of the normal saline. (F) Correlation coefficient between the spectrum of the supernatant of different washing times and the spectrum of the normal saline

In order to develop a convenient technique to quickly verify the quality of washing microbiota, NIRS spectroscopy was applied to analyze the changes of light intensity and absorbance after different times of washing. In this NIRS experiment, the normal saline was used as a standard control and its transmission light intensity was the highest, as well as the absorbance was the smallest as compared with other samples (Fig. 9). As the washing times increasing, the light intensity of the supernatant increased consistently (*P* < 0.001), which was getting closer to that of the normal saline. There was a significant difference in the light intensity between the Supernatant 1 and the Supernatant 3 (*P* < 0.001). When it comes to the absorbance of light, Supernatant 1 exhibited the greatest absorbance compared to the other groups in each donor. And the absorbance showed a declining trend with the increase of washing times, which was closer to that of the normal saline. After repeated centrifugation plus suspension based on the automatic purification system, both light intensity and absorbance of Supernatant 5 were close to those of the normal saline. The analysis based on the euclidean distance and correlation coefficient demonstrated that the difference of light intensity between the supernatant and saline was getting smaller as the washing times increasing (*P* < 0.001).

## Discussion

The present study from population evidence showed that washed microbiota preparation significantly decreased FMT-related AEs in UC and CD as compared with the manual preparation. Undigested food residues, fungi, parasite eggs and some small particles can be removed by the microfiltration system. The followed washing process washed out a certain bacterial fragments, metabolites, soluble molecules, and proteins. The above physical washing process contributed to the decreased rate of WMT-related AEs. Further animal experiments and *in vitro* differential screening supported the evidences for linking the clinical findings of the safety of FMT.

The washing microbiota method makes precise dose of fecal microbiota administration feasible. Up to now, most researches and consensuses recommend doctors to determine the therapeutic dose of microbiota based on the donor’s fecal weight (Mattila et al., 2012; Satokari et al., 2015; Costello et al., 2016). This study first time reported that the fecal weight was not well correlated with the amount of enriched microbiota. This phenomenon can be observed among donors of different ages, and even different donations in one same donor. In terms of therapeutic dose, many studies recommend a single dose of 30–50 g fecal material to treat patients (Mattila et al., 2012; Satokari et al., 2015; Costello et al., 2016). The latest international consensus in 2019 suggested that the minimum quantity of donor stool is 12.5 g for upper gastrointestinal delivery, and 25 g for lower gastrointestinal delivery (Cammarota et al., 2019). The consensus also proposed that the ideal dose using fecal weight for clinical treatment remains uncertainty (Cammarota et al., 2019). In our center, we delivered a precise dose of microbiota based on the amount of enriched bacteria. This protocol might be one of the key factors resulting in satisfactory clinical efficacy in indications beyond CDI. Notably, a higher speed and a longer time were not used in current experimental conditions for centrifuging and washing bacteria for potentially saving living bacteria.

From the animal experiment, we found that the Supernatant 1 caused 70% of death in mice during 16–24 h after intraperitoneal injection. The remaining 30% of mice were very weak with chills. Meanwhile, no death was observed in other groups. The mice injected with the Supernatant 3 and 4 had the mildest and similar toxic reaction at both 6 and 24 h after intraperitoneal injection, respectively. The peripheral blood indexes of the Supernatant 3 were found to be close to the normal saline group, including WBC, RBC, PLT, percentage of NEUT and LYM. Further analysis indicated that no significant difference in blood cell counts between the third and fourth supernatant was found. Four times of centrifugation plus suspension may not be needed. These results from animal experiments verified the protocol in our FMT center using three times of centrifugation plus suspension after microfiltration. Then, the Supernatant 1 and Supernatant 3 were used for differential studies. Notably, the Supernatant 1 obtained after microfiltration plus centrifugation should be reasonably better than that from the manually prepared fecal suspension after crude filtration by using sterile dressings or kitchen strainer. This experimental mouse model was designed to amplify the host response to materials by intraperitoneal injection. This might be a promising animal model for evaluating the toxicity of materials which might be not easily detected by delivering them through intestinal tract.

In the hematoxylin-eosin (HE) staining assay for the spleen of mice, the proliferation of germinal center in secondary lymphoid follicles was only observed in the group of Supernatant 1, which corresponds with its serious sepsis (Pilgrim et al., 2007). This immune response may be related to the stimulation of bacterial fragments and their metabolites, as well as some small soluble molecules in the supernatant. These materials can act as antigens leading to sepsis. The obvious NEUT infiltration was also observed in the group of Supernatant 1. The results further support that the toxic role of Supernatant 1 is stronger than Supernatant 3.

The number of WBC in the group of Supernatant 1 decreased significantly both at 6 h and 24 h after injection compared with the normal group of normal saline. These results indicated that the decline of WBC in mice might be a reflection of sepsis exacerbation in this study. This may be explained by the fact that these mice occur lipopolysaccharide (LPS)-induced endotoxemia and systemic inflammatory response syndrome (SIRS) after intraperitoneal injection with the fecal microbiota supernatant. In the fecal supernatant injected into mice there should be a certain bacteria fragments and their metabolites, small soluble molecules, as well as proteins washed out during the protocol for preparation of washed microbiota. As is known, Gram-negative bacteria account for a large percent of the bacteria in the gastrointestinal tract (Suffredini and Noveck, 2014). The fecal supernatant after repeated washing may play a toxic role in the host cells through the action of LPS from Gram-negative bacteria. LPS, a component of the outer wall of Gram-negative bacteria and also a kind of endotoxin, could activate a series of signal transduction pathways through the Toll-like receptors on the surface of the cell membrane to promote the occurrence of inflammation when it acted on the host cell (Jilma-Stohlawetz et al., 2017). Kurhaluk et al. established an endotoxemia model of mice (Kurhaluk et al., 2018) and observed an overall downward trend of WBC in the group of LPS. In addition, Yates et al. (2011) also found that in the LPS-induced ewes model, the number of WBC decreased significantly within 6 h after injection compared with the baseline. These reports are consistent with our findings in this study.

The group of Supernatant 1 showed a significantly decreased number of WBC and an increased percentage of NEUT at 24 h compared with that at 6 h. This change may explain the increased mortality of 70% in the group of Supernatant 1. The safety of the Supernatant 3 used in clinical actually was verified again in the mice model.

To investigate what was washed out after the last centrifugation, we performed metagenomic NGS analysis on the virus in the Supernatant 1 and Supernatant 3 from five healthy donors. As a result, we found a total of 34 types of viruses. Interestingly, all of these detected viruses (Aboul-Ata et al., 2014; Symonds et al., 2018) from donors might be left-over from plant-based food. However, the previous researches in virome are limited. The further identification should be performed in the future to confirm whether these viruses were from human or not. The type and sequencing read of the virus were found to be different among the five donors, suggesting a possible role for the diet. Only one virus named pepper mild mottle virus was present in all five donors, and its sequencing reads increased consistently in the Supernatant 3 as compared with the Supernatant 1. Pepper mild mottle virus as a plant pathogen might be a useful index virus for enteric viruses in monitoring the microbial quality of fresh produce and shellfish (Symonds et al., 2018). Similarly, Tobacco mild green mosaic virus was present in four donors, and its sequencing reads were also elevated in the Supernatant 3. Tobacco mild green mosaic virus coat protein could be used as an expression vector for the mimotope to be expressed into tobacco plants. Then this expressed recombinant protein, as an edible vaccine, had both a therapeutic and a diagnostic role (Aboul-Ata et al., 2014). Consistently, the total viral types and sequencing number of the five donors increased in the Supernatant 3 as compared with the Supernatant 1. The results indicate the viruses could be eluted from the microbiota into the supernatant in normal saline as a vector solution, especially for Watermelon mosaic virus, Cherry green ring mottle virus and Paprika mild mottle virus.

The differential metabolites with pro-inflammatory effects were found to be mainly washed out in the Supernatant 1, including LtB4, CORT, PGG2, and 5-HIAA. Kwon et al. (2019) found that LtB4 and its receptors (BLT1 and BLT2) acting as inflammatory lipid mediators aggravated LPS-induced endotoxic shock in mice. CORT was (Lin et al., 2019) confirmed to be involved in the neuroinflammatory response in the LPS-induced sepsis model. As is known to all, prostaglandins (PGs) as a metabolite of arachidonic acid play an important role in fever, inflammation and blood pressure regulation. PGG2 was an intermediate metabolite which can be further transferred into different PGs (such as PGI2, PGE2, PGF2α, PGD2) through prostaglandin synthase (Alhouayek and Muccioli, 2014). The increased PGG2 can increase pro-inflammatory macrophage activation induced by LPS *in vitro* (Alhouayek et al., 2013). The concentration of 5-HIAA was reported to be associated with highly sensitive C-reactive protein, which was a marker of chronic low-grade inflammation in metabolic syndrome (Afarideh et al., 2015). Hence, the presence of these pro-inflammatory substances may explain the aggravation of inflammation and even death in mice injected with the Supernatant 1. Meanwhile, several significantly enriched metabolic pathways played an important role in the inflammatory reaction. The pathway of inflammatory mediator regulation of TRP channels plays different roles in inflammatory response due to it consists of more than 30 members which can be divided into 7 subfamilies (TRPC, TRPV, TRPM, TRPA, TRPP, TRPML, TRPN). TRPMs can inhibit the secretion of anti-inflammatory cytokine (Nilius and Owsianik, 2011) and participate in the inflammatory process through the antagonism against neutrophils (Wang et al., 2014). Boltana et al. (2018) found that TRPVs participated in the process of temperature up-regulation concomitantly with other pro-inflammatory cytokines such as PGE2, TNF-α, IL-6, and IL-1β.

In Supernatant 3, a total of 13 anti-inflammatory metabolites were noted. Many of them can inhibit the activation of the LPS-induced NF-κB signaling pathway to reduce the inflammatory response, including tretinoin (Austenaa et al., 2009), stearidonic acid (Sung et al., 2017), caffeic acid (Kim et al., 2014), tyrosol (Lu et al., 2013), aspirin (Liu et al., 2017), 4-hydroxy-2-nonenal (Kim et al., 2009). Resveratrol (Bigagli et al., 2017), chloral hydrate (Cai et al., 2016) and paracetamol (Vuong et al., 2019) have anti-inflammatory and anti-oxidant effects in LPS-stimulated RAW 264.7 macrophage cells *in vitro*. Calcitriol, the active form of vitamin D, has been confirmed that it can reduce the infiltration of inflammatory cells and attenuate the elevation of TNF-α during LPS-induced acute lung injury in mice (Tan et al., 2016). Similarly, in the lung injury model of mice (Metsola et al., 2014), adenosine regulates endothelial permeability and plays a role of anti-inflammation via its receptors. These anti-inflammatory metabolites increased in the Supernatant 3 may explain why the Supernatant 3 induced inflammation was less than that of the Supernatant 1. This should contribute to decreasing the rate of clinical AEs of microbiota transplant. Additionally, the process of washing microbiota does loss some beneficial molecules, such as anti-inflammation or anti-oxidant elements, the efficacy was not affected in UC (Ding et al., 2019) and CD (Wang et al., 2018).

The experiment using NIRS is a way to seek the standard of using the optical spectrum technique to control the quality of the washing process. The qualified final washed samples are based on the standard process. However, further study is necessary to determine whether this method can be widely used in practice.

There are some limitations in this study. More inflammatory markers tested in the mouse experiment should be more solid. In the metagenomic NGS for virus screening, not all genomes are available. The differential screening for enriched microbiota would be supportive of clarifying the value of washed microbiota preparation protocol.

In conclusion, this study first time provides the evidence from clinical findings, animal experiments and *in vitro* tests to support that the protocol of WMT is better than the manual preparation of FMT in improving safety, enriching the precise amount of microbiota and quality controllable in practice. Our findings might encourage more researches to use this novel preparation to enrich microbiota from feces in laboratory study and move the crude FMT into the stage of WMT in practice.

## Materials and methods

### Data analysis on FMT-related AEs in IBD

The potential factors related to AEs were analyzed in patients with IBD who underwent FMT (Fig. 1). All data were from CMTS (www.fmtbank.org), which was supported by China National Clinical Research Center for Digestive Diseases (Xi’an) for a long-term evaluation on the decision, treatment, efficacy and safety of microbiota transplant. The data related to FMT was collected from Oct 25, 2012 to Oct 10, 2019 by searching the database, which follows the ethical protocol of CMTS. The clinical data of this clinical study was based on our pooled registered trials (NCT01790061, NCT01793831 and NCT02998112) in clinicaltrails.gov. AEs were confirmed by the international guideline Common Terminology Criteria for Adverse Events (CTCAE) version 5.0 as our previous reports (Wang et al., 2018; Ding et al., 2019). All trials were approved by the institutional review board of the Second Affiliated Hospital of Nanjing Medical University and the animal ethics committee of Nanjing Medical University. Written informed consent was obtained from all subjects.

### Fecal microbiota preparation

All recorded data on the weight of donated feces, the time for donation and the age of donors were collected for analysis (Fig. 1). Donors were recruited according to the long-term criteria for donor selection at our center (Ding et al., 2019; Zhang et al., 2019). Briefly, we used the eight criteria for screening a donor, including age, physiology, pathology, psychology, honesty, time, environment and recipient state (Ding et al., 2019; Zhang et al., 2019).

The method for preparation of microbiota is based on the automatic microbiota purification system followed with centrifugation plus suspension for three times in a specially designed exclusive laboratory at good manufacture practice (GMP) level (Cui et al., 2016; Zhang et al., 2018). The fecal particles, parasites eggs and fungus in the fecal suspension are removed by sequential microfiltration based on the automatic machine. The “one-hour FMT protocol” limited the time from defecation of a donor, laboratory preparation for enriching microbiota to the time of microbiota delivering, or microbiota storing within one hour (He et al., 2017; Zhang et al., 2018; Ding et al., 2019). The donated feces were collected using a disposable bottle matching the GenFMTer. The ratio of 500 mL saline per 100 g feces was used for homogeneously making fecal suspension, and then the scheduled microfiltration within the machine was automatically processed. The fecal microbiota suspension after microfiltration was automatically dispersed into serial 50-mL tubes for the following centrifugation for 3 min at a speed of 700 ×*g* (2000 rpm, TDZ5-WS, XIANGZHI, Changsha, China). The supernatant obtained after centrifugation of the fecal microbiota suspension was suctioned. The same subsequent centrifugation was performed after 0.9% sodium chloride was added to the microbiota precipitation. Briefly, the fecal supernatant after the first to fifth centrifugation plus suspension was collected for animal experiments or metabolism and virus tests. The fecal supernatant obtained after filtration from GenFMTer following the first centrifugation was defined as Supernatant 1. The fecal supernatant after the repeated centrifugation plus suspension for two, three, four and five times was defined as Supernatant 2, Supernatant 3, Supernatant 4, and Supernatant 5, respectively. The study flow was shown in Fig. 1.

### Mouse model by intraperitoneal injection

All experimental protocols were reviewed and approved by the animal ethics committee of Nanjing Medical University (No. IACUC-1910004). The male C57BL/6 mice weighing 17–20 g from Animal Center of Nanjing Medical University were used. The mice were injected intraperitoneally with the fecal supernatant at the dose of 0.3 mL per mouse. The control group was injected with the same dose of 0.9% sodium chloride. The observation points are set at 6 h after injection, time of death, and 24 h after injection if still alive. We took peripheral blood from the orbit for routine analysis of blood and pathological sections of the spleen to observe the infiltration of inflammatory cells. Blood samples from retro-orbital plexus were collected in an anticoagulant tube containing EDTAK2 (KANG JIAN, China) and analyzed using Automatic Hematology Analyzer (Hisenmekang-XN550, Japan). No significant differences in sepsis of mice were observed between Supernatant 3 and Supernatant 4 as well as Supernatant 5. Then, the Supernatant 1, Supernatant 2, Supernatant 3 and Supernatant 4 were selected for the next experiments. The spleen tissues were taken and soaked in 4% paraformaldehyde for a paraffin slice and HE staining. Neutrophil infiltration and proliferation of follicles were evaluated and graded. The number of neutrophils in the visual field differed from scattered to plentiful was classified as “+-+++”. “+” means scattered neutrophils, while “++” or “+++” means moderate and plentiful number of neutrophils, respectively. Follicles included primary follicles and the secondary follicles. The classification of the follicles proliferation was none or existing. These experiments further confirmed that the Supernatant 1 and Supernatant 3 were collected for further differential screening on viruses and metabolites.

### Virus screening by metagenomic NGS

Total 1.5 mL of Supernatant 1 and 3 were collected for nucleotides purification with TIANamp Micro DNA Kit (DP316, TIANGEN BIOTECH) for DNA extraction and Vision Medicals Cat (VM001, Guangzhou, China) for RNA extraction according to the manufacturer’s recommendation (Miller et al., 2019). DNA was sheared under power 50 for 155 s by using focused-ultrasonicators (Covaris). In total, 100 ng sheared DNA was subjected to library construction with VAHTS^TM^ Universal DNA Library Prep kit (ND 607, VAZYME BIOTECH). After purification and sorting, Agilent 2100 bioanalyzer was used to profile the DNA length of the library, which was purified and sorted. Library with 300 ± 50 bp peak passed and was subjected to library pooling. Before being subjected to library preparation, concentrations of extracted DNA/RNA were measured by a Qubit Fluorometer. Human rRNA molecules from the RNA samples were depleted by an RNase H-based method (Vision Medicals Cat# VM003, Guangzhou, China). DNA library was prepared by a transposase-based methodology.

Illumina NextSeq550 sequencers were used to sequence by using a 75 bp, single-end and single index sequencing kit (Illumina Cat#CN500, San Diego). Each sample can obtain approximately 20 million reads. High-quality sequencing data were generated after excluding low-quality and short (length < 35 bp) reads, and human host sequences were subtracted, which were mapped to the human reference genome (hg39) and plasmids using Burrows-Wheeler alignment (Li and Durbin, 2009). After the removal of low complexity reads, the remaining data were mapped to the IDseq microbial genome databases consisting of viruses, bacteria, fungi, and parasites. The taxonomic references were downloaded from the National Center Biotechnology Information (ftp://ftp.ncbi.nlm.nih.gov/genomes/ftp://ftp.ncbi.nlm.nih.gov/genomes/). Upon identification of critical pathogen, the identified species-specific sequences were further confirmed by Blastn to validate its accuracy (http://www.blast.ncbi.nlm.nih.gov/Blast.cgi). The experiment was performed by Vision Medicals company (Guangzhou, China).

### Metabolism analysis by LC-MS/MS

The supernatant samples were stored at −80 °C before being sent to the company (BGI, Wuhan) for analysis. Methanol was used for extraction for MS analysis. After repeated grinding (50 Hz, 5 min) and centrifuging (25,000 rpm, 15 min), metabolites were extracted for liquid chromatography-mass spectrometry analysis. Waters 2D UPLC (Waters, USA) tandem Q Exactive high-resolution mass spectrometer (Thermo Fisher Scientific, USA) was used for the separation and detection of metabolites. The chromatographic column used was BEHC18 column (1.7 μm 2.1 × 100 mm, Waters, USA). The running mode of LC-MS was called the binary gradient solvent mode, consisting of 0.1% (*v*/*v*) formic acid in water (solvent A) and 0.1% formic acid in 100% methanol (solvent B) in the positive ion mode and 10 mmol/L formic acid ammonia in water (solvent A) and 10 mmol/L formic acid ammonia in 95% methanol (solvent B) in negative ion mode. We used HMDB, KEGG and LipidMaps database to analyze the data.

### Spectrum analysis

In order to quickly confirm the quality of the washing process for enriching microbiota from feces, NIRS was used to perform the differential test for each supernatant after centrifugation. The QE Pro high-performance spectrometer (Ocean Optics Inc. USA) and HL-2000-LL light source (Ocean Optics Inc. USA) were used to setup the measurement system. The effective detection wavelength range was set as 300–950 nm. Solution sample were added into a cuvette for tests. Each sample was measured for 10 times, repeatedly, to verify the stability of the experimental system. The spectral curves of each sample would be obtained for analysis. The software-supported system was set by the Biomedical Engineering Department, TianGong University, China.

### Statistical analysis

Data were analyzed and performed using SPSS (Chicago, IL, USA) and GraphPad Prism (La Jolla, CA, USA). When the normality of the distribution of variables was acceptable, the unpaired Student’s *t*-test and one-way ANOVA were used to analyze differences between groups. Otherwise, the nonparametric test was used. Categorical variables were analyzed by chi-square test. Pearson correlation was used for correlation analysis. Differences were considered significant when *P* < 0.05. The q value is obtained after the false discovery rate (FDR) correction of the *P* value.

## Electronic supplementary material

Below is the link to the electronic supplementary material.

Supplementary material 1 (PDF 748 kb)

## Abbreviations

AE: adverse event
CDI: *Clostridioides difficile* infection
CD: Crohn’s disease
CORT: corticosterone
CMTS: China Microbiota Transplantation System
FDR: false discovery rate
FMT: fecal microbiota transplantation
GMP: good manufacture practice
KEGG: Kyoto encyclopedia of genes and genomes
LYM: lymphocyte
LtB4: leukotriene B4
LPS: lipopolysaccharide
NGS: next-generation sequencing
NIRS: near-infrared absorption spectroscopy
NEUT: neutrophil
NLR: NEUT to LYM ratio
PLT: platelet
PLR: PLT to LYM ratio
PCA: principle component analysis
PGs: prostaglandins
PLS-DA: partial least squares method-discriminant analysis
PGG2: prostaglandin G2
RBC: red blood cell
RCT: randomized controlled trial
SIRS: systemic inflammatory response syndrome
TRP: transient receptor potential
UC: ulcerative colitis
WBC: white blood cell
WMT: washed microbiota transplantation
