# Supplementary material for: Washed microbiota transplantation vs. manual fecal microbiota transplantation: clinical findings, animal studies and in vitro screening
Source: Protein Cell. 2020 Jan 9;11(4):251–66. doi: 10.1007/s13238-019-00684-8 (PMC7093410; doi:10.1007/s13238-019-00684-8)
Supplement: Supplementary file 1 — Supplementary material 1 (PDF 748 kb) [file 13238_2019_684_MOESM1_ESM.pdf]

Supplementary files

Supplementary Table 1. Rate of AEs in patients with IBD.

AEs	Manual	Automation
Fever	6% (6/100)	3.68% (32/870)
Increased stool frequency	14% (14/100)	2.99% (26/870)
Abdominal pain	4% (4/100)	1.38% (12/870)
Abdominal bloating	2% (2/100)	0.11% (1/870)
Pruritus	0% (0/100)	0.69% (6/870)
Nausea/Vomiting	0% (0/100)	0.92% (8/870)
Frequent exhaust	3% (3/100)	0% (0/870)
Herpes zoster	0% (0/100)	0.11% (1/870)

Supplementary Table 2.

	**The preparation of fresh washed microbiota suspension**
(1)	Feces are collected on site in a specific disposable feces container in the dedicated room only for donor
(2)	All devices directly contacting fecal matters used for the fecal collection, suspension filtration, centrifugation and washing should be disposable
(3)	The weight of donated feces less than 50g from adult donor is not recommend to enter the process for avoiding the consumption of disposable devices
(4)	All collected feces are put into the process of automatic purification system for enriching microbiota
(5)	The fecal suspension is transferred to centrifuge tubes for centrifugation with

	700×g (2000 rpm, TDZ5-WS, XIANGZHI, Changsha, China) for 3 minutes and then discard most supernatant
(6)	This is repeated for 3 times by adding sterile saline for making suspension
(7)	10 cm^3 (~1 × 10^{13} bacteria) of final precipitated microbiota as the basic dose unit for clinical use. The volume ratio of final precipitation/vector solution is 1:2 for making suspension as fresh use or frozen use

Supplementary Figure 1. Changes of peripheral blood cells in the five groups of mice after 6 hours of intraperitoneal injection of fecal microbiota supernatant. (A) Changes in the percentage of NEUT (n = 8 animals/group). (B) Changes of NEUT (n=8 animals/group). (C) Changes of NLR (n = 8 animals/group). (D) Changes in the percentage of LYM (n = 8 animals/group). (E) Changes of LYM (n = 8 animals/group). (F) Changes of PLR (n=8 animals/group). Statistical comparisons are performed using one-way ANOVA; *P < 0.05, **P < 0.01, ***P < 0.001. Data are presented as mean ± SD.

Supplementary Figure 2. Changes of peripheral blood cells in the five groups of mice after 24 hours of intraperitoneal injection of fecal supernatant. (A) Changes in the percentage of NEUT (Supernatant 1, n = 3; Other groups, n = 8). (B) Changes of NEUT (Supernatant 1, n = 3; Other groups, n = 8). (C) Changes of NLR (Supernatant 1, n = 3; Other groups, n = 8). (D) Changes in the percentage of LYM

(Supernatant 1, n = 3; Other groups, n = 8). (E) Changes of LYM (Supernatant 1, n = 3; Other groups, n = 8). (F) Changes of PLR (Supernatant 1, n = 3; Other groups, n = 8). Statistical comparisons are performed using one-way ANOVA; *P < 0.05, **P < 0.01, ***P < 0.001. Data are presented as mean ± SD.

Supplementary Figure 3. Changes of peripheral blood cells at 6 hours and 24 hours after intraperitoneal injection of Supernatant 1 and Supernatant 3. (A) Changes of NEUT (Supernatant 1, 6 hours, n = 8; Supernatant 1, 24 hours, n=3). (B) Changes in the percentage of LYM (Supernatant 1, 6 hours, n=8; Supernatant 1, 24 hours, n=3). (C) Changes of LYM (Supernatant 1, 6 hours, n=8; Supernatant 1, 24 hours, n=3). (D) Changes of NEUT (Supernatant 3, 6 hours /24 hours, n=8). (E) Changes in the percentage of LYM (Supernatant 3, 6 hours /24 hours, n=8). (F) Changes of LYM (Supernatant 3, 6 hours /24 hours, n=8). Statistical comparisons are performed using unpaired t-tests; *P < 0.05, **P < 0.01, ***P < 0.001. Data are presented as mean ± SD.

Supplementary Figure 4. The light intensity of fecal microbiota supernatant by near-infrared absorption spectroscopy.

Supplementary Figure 5. The absorbance of fecal microbiota supernatant by near-infrared absorption spectroscopy.

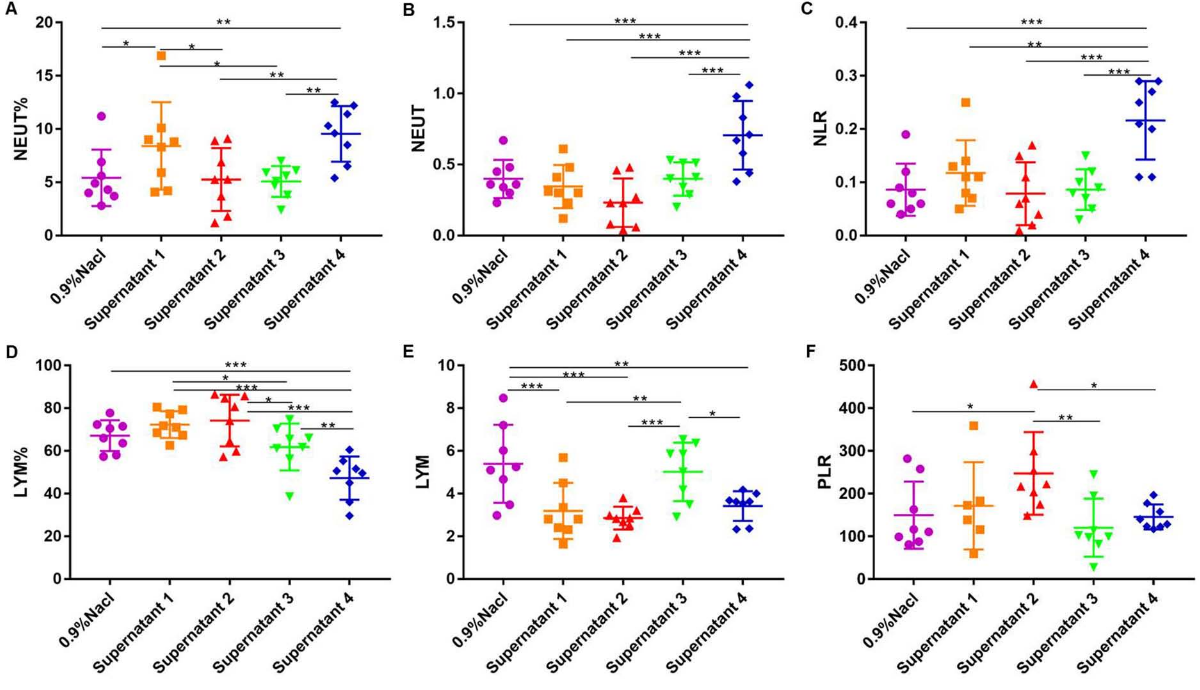

A
NEUT%
B
NEUT
C
NLR
D
LYM%
E
LYM
F
PLR
0.9%Nacl
Supernatant 1
Supernatant 2
Supernatant 3
Supernatant 4

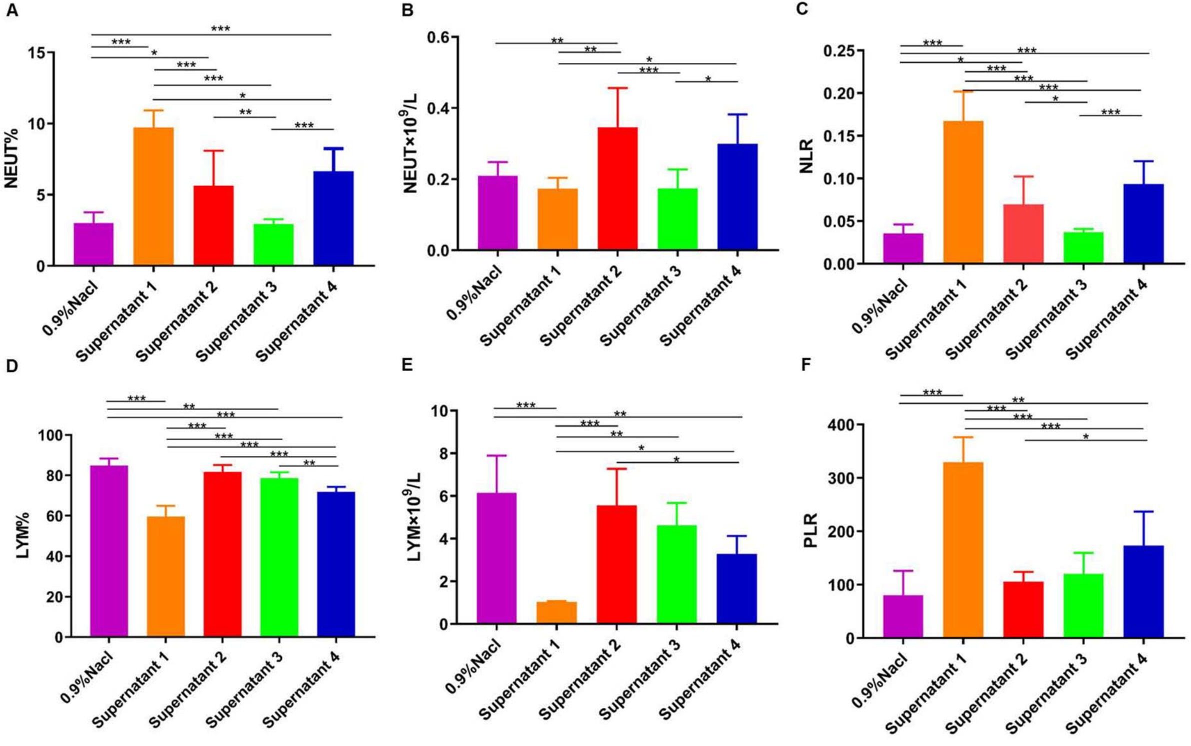
A
NEUT%
0.9%Nacl
Supernatant 1
Supernatant 2
Supernatant 3
Supernatant 4
B
NEUT×10⁹/L
C
NLR
D
LYM%
E
LYM×10⁹/L
F
PLR

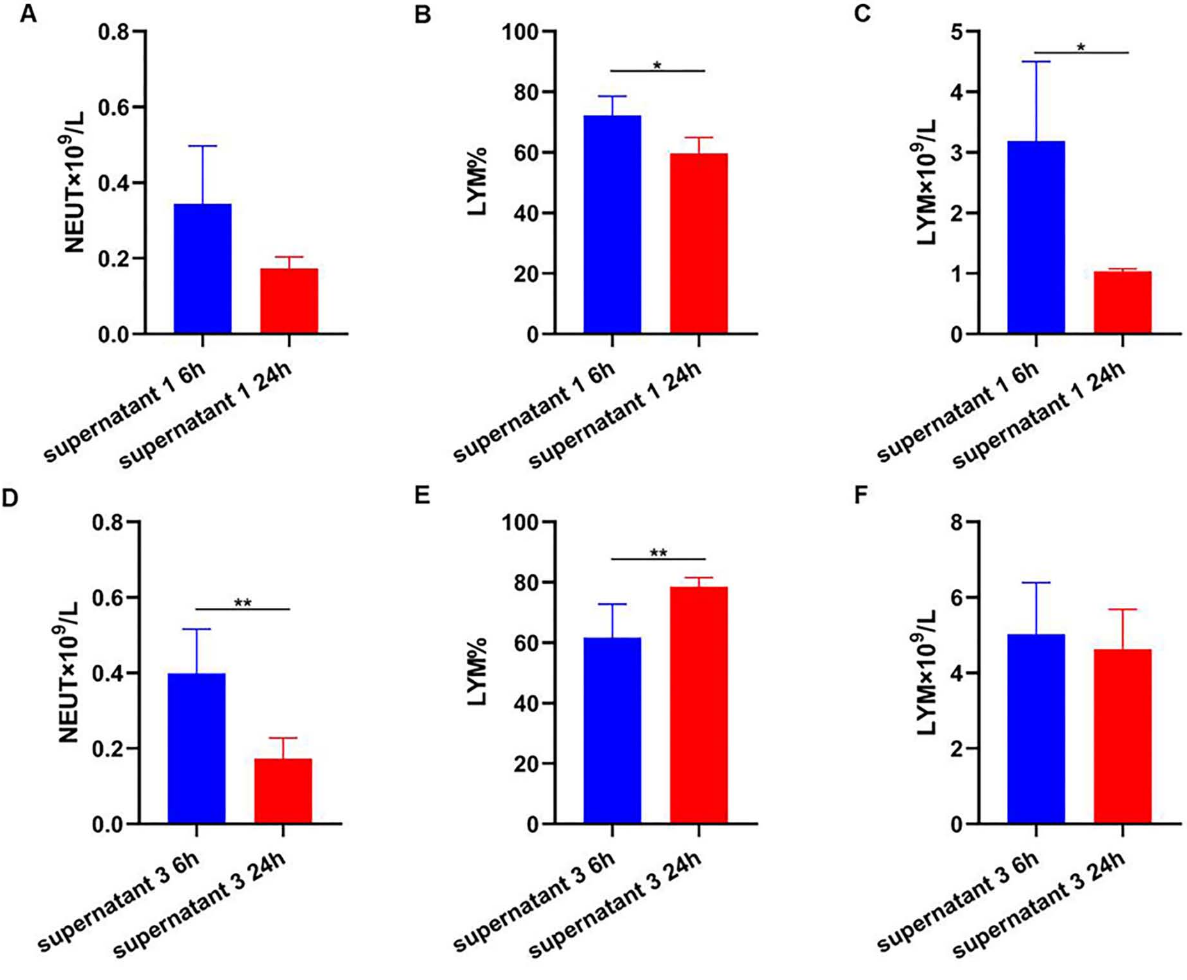
A
B
C
D
E
F
NEUT×10⁹/L
LYM%
LYM×10⁹/L
*
**
supernatant 1 6h
supernatant 1 24h
supernatant 3 6h
supernatant 3 24h

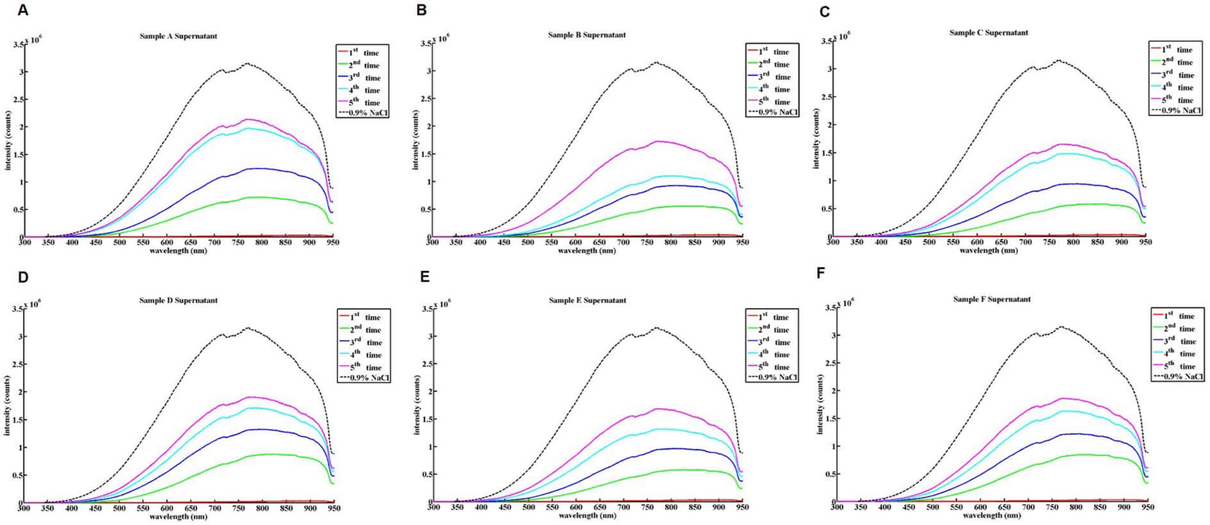
A
Sample A Supernatant
B
Sample B Supernatant
C
Sample C Supernatant
D
Sample D Supernatant
E
Sample E Supernatant
F
Sample F Supernatant
x 10^6
intensity (counts)
wavelength (nm)
1st time
2nd time
3rd time
4th time
5th time
0.9% NaCl

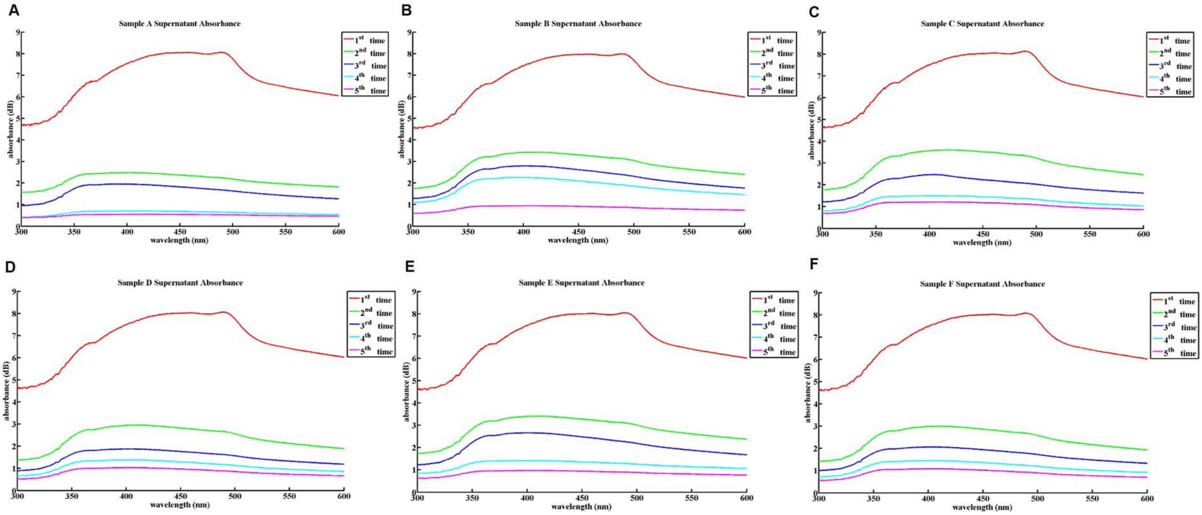
A
Sample A Supernatant Absorbance
B
Sample B Supernatant Absorbance
C
Sample C Supernatant Absorbance
D
Sample D Supernatant Absorbance
E
Sample E Supernatant Absorbance
F
Sample F Supernatant Absorbance
1st time
2nd time
3rd time
4th time
5th time
absorbance (dB)
wavelength (nm)
